# Synergistic Defect and Phase Boundary Engineering for Large Strain and Superior Low-Field Energy Storage in Bi_0.5_Na_0.5_TiO_3_-Based Relaxors

**DOI:** 10.3390/ma19112328

**Published:** 2026-06-01

**Authors:** Hui Li, Zhongfeng Shang, Xiaojun Ren, Wenfang Li, Shengguo Gao, Tengfei Zhang, Pingyuan Liu, Zongshuai Shao, Yangyang Zhang

**Affiliations:** 1 Faculty of Engineering, Huanghe Science and Technology College, Zhengzhou 450063, China; leehui@hhstu.edu.cn (H.L.); 18768873383@163.com (X.R.); 18236982569@163.com (W.L.); 18239471423@189.cn (T.Z.); 17538311732@163.com (P.L.); 19712957601@163.com (Z.S.); 2 Hanwei Electronics Group Corporation, Zhengzhou 450001, China; 3 College of New Energy, Zhengzhou University of Light Industry, Zhengzhou 450002, China; 4 Zhengzhou Winsen Electronics Technology Company Limited, Zhengzhou 450001, China; gaosg@winsensor.com

**Keywords:** BNT-based relaxors, large strain, energy storage, lead-free ferroelectrics, multifunctional materials

## Abstract

**Highlights:**

- The single dopant enables synergistic defect and phase boundary engineering for multifunctional BNT-based ceramics.
- ST substitution stabilizes the ER state and shifts the NR-ER transition toward room temperature.
- The composition with *x* = 0.15 delivered a large strain of 0.45% and a reduced strain hysteresis of 10.8%.
- Superior low-field energy storage properties (*W*_rec_ = 1.06 J/cm^3^, *W*_rec_/*E* = 0.013 mC/cm^2^, *η* = 81%) were realized at *x* = 0.25.

**Abstract:**

The advancement of microelectromechanical systems (MEMS) drives the demand for multifunctional ferroelectrics that synergistically combine substantial strain with competitive energy storage capabilities. In this work, the simultaneous enhancement of electromechanical strain and energy storage properties is achieved in (1−*x*)(Bi_0.5_Na_0.5_)_0.94_Ba_0.06_(Ti_0.98_Mn_0.02_)O_3_-*x*SrTiO_3_ (0 ≤ *x* ≤ 0.3) ceramics by synergistically employing A-site defect engineering and the nonergodic/ergodic relaxor (NR/ER) phase boundary design. The incorporation of Sr^2+^ plays a dual role: it induces cationic disorder that expands the polarization difference (Δ*P = P_max_* − *P_r_*), thereby effectively boosting the recoverable energy density (*W*_rec_). Concurrently, it stabilizes a critical NR/ER phase ratio near room temperature, which maximizes the strain while minimizing the strain hysteresis. Consequently, when *x* = 0.15, the optimized system delivers a large strain of 0.45% (*d*_33_* = 562 pm/V) with low hysteresis (*H* = 10.8%). In addition, the *x* = 0.25 composition exhibits an enhanced *W*_rec_ of 1.06 J/cm^3^, a competitive energy-storage potential (*W*_rec_/*E*) of 0.013 mC/cm^2^, and a high efficiency (*η*) of 81% under 80 kV/cm. This work provides an effective strategy for developing multifunctional lead-free materials for integrated actuators and energy storage devices.

## 1. Introduction

As microelectromechanical systems (MEMS) technology matures, there is a growing demand for multifunctional materials. Among these, ferroelectrics possessing a giant strain response alongside high energy-storage density have attracted significant research interest [1,2,3]. To date, lead-based ferroelectrics dominate commercial applications in precision actuators and energy storage capacitors owing to their superior performance in large electromechanical strain and high recoverable energy storage density (*W*_rec_). For instance, Dy-doped (PbBL)(ZrSnT)O_3_ multilayer ceramic capacitors achieve an exceptionally large strain of 0.71%, coupled with an energy density of 2.7 J/cm^3^ at 300 kV/cm [4]. Concurrently, (Pb,Sm)(Zr,Sn,Ti)O_3_ multifunctional ceramics simultaneously deliver a giant field-induced strain of 0.63% alongside a superior *W*_rec_ of 1.743 J/cm^3^ [5]. However, ecologically suitable lead-free alternatives are imperative due to growing environmental and safety concerns. In recent years, Bi_0.5_Na_0.5_TiO_3_ (BNT)-based relaxor ferroelectrics (RFEs) have shown considerable potential in field-induced strain and energy storage owing to their complex phase transition process and desired polarization features, characterized by a minimized remanent polarization (*P*_r_) and a maximized polarization (*P*_max_) through composition design [6,7]. For instance, MnO-doped 0.65BNT-0.35ST RFEs have demonstrated a high *W*_rec_ of 1.14 J/cm^3^ with an energy efficiency (*η*) of 83%, as well as a strain of 0.22% with low-strain hysteresis (*H*) of 14% under 89 kV/cm [8]. The ergodic relaxor BNT-0.25SLBT has been reported to deliver a large strain response of 0.47% with an *H* of 25% and a super-high *W*_rec_ of 3.18 J/cm^3^ with *η* = 82.8% under 250 kV/cm [9]. Nevertheless, current BNT-based materials still underperform compared to their lead-based counterparts. Furthermore, while most studies have focused on either large strain or high energy density individually, the simultaneous enhancement of both properties remains a significant challenge for practical multifunctional applications.

For actuators, high strain response and low strain hysteresis are two critical performance metrics. The former is required to achieve large actuation strokes, and the latter is crucial for maintaining operational precision and ensuring long-term reliability by minimizing energy loss [10]. However, previous studies have revealed an inherent trade-off between these two parameters. For instance, the Bi_0.5_Na_0.5_TiO_3_-BaTiO_3_-K_0.5_Na_0.5_NbO_3_ system achieves a high strain of 0.45% but is accompanied by high hysteresis (*H* = 68.6%) [11]. Conversely, systems with low hysteresis (*H* < 10%) are often limited to a modest strain of about 0.2% [12]. Therefore, the simultaneous achievement of high-strain (>0.4%) and low-strain hysteresis (*H* < 10%) under practical electric fields remains a significant challenge in BNT-based materials. For dielectric capacitors, synergistic optimization of *W*_rec_ and *η* is crucial. A high *W*_rec_ enables greater energy storage per unit volume, which is key to the miniaturization of electronic devices. Meanwhile, a high *η* implies lower energy loss, thereby enhancing device reliability and longer service life. In recent years, numerous studies have reported excellent energy storage properties in BNT-based ceramics. For instance, the (BNT-BT)-0.25CBST ceramic achieves an ultrahigh *W*_rec_ of 12.2 J/cm^3^ with a high *η* of 88.8% under a high electric field of 660 kV/cm. However, the high energy storage performance deteriorates significantly under reduced fields, with *W*_rec_ dropping sharply to 0.74 J/cm^3^ at 100 kV/cm [13]. As a pivotal metric for evaluating energy-storage applications, the energy-storage potential (*W*_rec_/*E*) quantifies the recoverable energy density per unit field, where a high value is essential for enhancing device reliability and facilitating miniaturization [14,15]. The heavy reliance on high electric fields (HEFs > 200 kV/cm) not only increases the risk of device failure but also conflicts with the low-voltage operational requirements of modern electronics like integrated circuits and wearable devices [16,17]. Therefore, developing materials that maximize *W*_rec_/*E* to simultaneously achieve superior *W*_rec_ and *η* at low electric fields has become an urgent technological imperative for ensuring energy storage device reliability, safety, and integration. Consequently, synergistically optimizing these properties—integrating large strain and low *H* with superior *W*_rec_ and *η* under low electric fields—represents a critical step toward realizing advanced multifunctional applications.

The morphotropic phase boundary (MPB) composition 94(Bi_0.5_Na_0.5_)TiO_3_-6BaTiO_3_ (94BNT-6BT) exhibits large strain and a superior *P*_max_ at low electric fields, owing to the comparable free energy between its rhombohedral and tetragonal phases [18,19]. These characteristics make it a promising candidate for multifunctional devices requiring concurrent large strain and high energy density. However, the substantial *H* and high *P*_r_ significantly undermine the overall performance, representing a formidable barrier to practical applications. Previous work indicates that Mn doping introduces defect dipoles, which establish a stable internal bias field that not only facilitates ferroelectric domain switching for large strain but also provides a restoring force upon field removal to drive domain recovery, thereby enhancing strain with reduced hysteresis [20]. Despite the benefits of defect engineering via Mn doping, the strain and energy storage performance in Mn-modified 94BNT-6BT systems remains inadequate for advanced actuators and capacitor applications. To concurrently address the challenges of excessive *H*, inadequate strain, and low *W*_rec_, we propose a strategy combining A-site defect engineering and nonergodic/ergodic relaxor (NR/ER) phase boundaries design. Taking advantage of its established MPB characteristics, we strategically employed (Bi_0.5_Na_0.5_)_0.94_Ba_0.06_(Ti_0.98_Mn_0.02_)O_3_ (BNBTM) as the matrix to incorporate functional defect dipoles. Building upon this basis, SrTiO_3_ (ST) was incorporated into the BNBTM matrix following a two-fold strategy. First, guided by phase boundary engineering, ST substitution shifts the nonergodic-to-ergodic relaxor (NR-to-ER) transition down to room temperature [21], inducing a critical NR/ER phase coexistence that optimizes the trade-off between large strain and low *H* [22]. Simultaneously, as proposed in analogous studies [20], Sr is expected to act as an A-site dopant to intensify cationic disorder and introduce supplementary defect dipoles. These microstructural modifications promote the formation of pinched polarization–electric field (*P*-*E*) hysteresis loops characterized by high *P_max_* and low *P*_r_, which are essential for superior energy storage. As a result of this deliberate compositional tuning, the (1−*x*)BNBTM-*x*ST system delivers a high strain of 0.45% (*d*_33_* = 562 pm/V) with a reduced *H* of 10.8% at *x* = 0.15. For *x* = 0.25, it exhibits a *W*_rec_ of 1.06 J/cm^3^, a *W*_rec_/*E* of 0.013 mC/cm^2^, and an *η* of 81% under a low electric field of 80 kV/cm. These findings provide a viable strategy for engineering multifunctional ferroelectrics that synergize large strain with high energy density.

## 2. Materials and Methods

### 2.1. Preparation of BNBMT-xST Ceramics

The (1−*x*)(Bi_0.5_Na_0.5_)_0.94_Ba_0.06_(Ti_0.98_Mn_0.02_)O_3_-*x*SrTiO_3_ ceramics with *x* = 0, 0.10, 0.15, 0.20, 0.25, 0.30 (molar ratio) were fabricated through a conventional solid-state sintering route. Stoichiometric mixtures of Na_2_CO_3_ (99.8%, Sinopharm, Shanghai, China), Bi_2_O_3_ (99%, Sinopharm, Shanghai, China), TiO_2_ (99%, Sinopharm, Shanghai, China), BaCO_3_ (99.9%, Sinopharm, Shanghai, China), MnO_2_ (99.5%, Sinopharm, Shanghai, China) and SrCO_3_ (99%, Sinopharm, Shanghai, China) underwent an initial 12 h ball-milling process in an ethanol medium, followed by oven-drying at 100 °C and subsequent calcination at 850 °C for 2 h. After a second ball-milling and drying, the powders were homogenized with a polyvinyl alcohol (PVA, Sinopharm, Shanghai, China) binder and shaped into green disks approximately 1 mm thick. To ensure complete elimination of the temporary PVA agent, the specimens underwent a low-temperature debinding profile, and were subsequently densified via sintering at 1160 °C for 2 h. Finally, the matured ceramic disks were surface-polished and metallized with silver pastes on both parallel faces to facilitate subsequent electrical characterizations.

### 2.2. Characterization

A ferroelectric analyzer (LC II-100V, Radiant Technologies Inc., Burbank, CA, USA) was used to measure *P*-*E* hysteresis loops, while a laser displacement sensor recorded the strain–electric field (*S*-*E*) curves. X-ray diffraction (XRD,Panalytical B.V., Almelo, Netherlands) utilizing Co Kalpha radiation was performed under ambient conditions for phase identification. The grain morphology and microstructure of the sintered samples were characterized using scanning electron microscopy (SEM, SU8010, Hitachi Ltd., Tokyo, Japan). An LCR meter (TH2827, Tonghui Electronic Co., Ltd., Changzhou, China) was used to collect dielectric data. A quasi-static *d*_33_ testing apparatus (ZJ-3AN, Chinese Academy of Sciences, Beijing, China) was employed to determine the piezoelectric constant (*d*_33_) of the poled samples. For this measurement, samples were first poled at 20 °C in a silicone oil (4 kV, 20 min) and then aged at room temperature for 48 h. Other measurements were conducted on unpoled ceramics. The normalized strain *d*_33_* (*d*_33_* = *S*_max_/*E*_max_) and *H* (*H* = Δ*S*_max/2_/*S*_max_, where Δ*S*_max_/2 is the strain difference at *E*_max_/2) were extracted from unipolar *S*-*E* curves. The energy storage properties (*W*_total_ = $\int_{0}^{p_{m}}\mathrm{Edp}$, *W*_rec_ = $\int_{p_{r}}^{p_{m}}\mathrm{Edp}$, and *η* = $W_{rec}/W_{total}$) were determined from the *P*-*E* hysteresis loops.

## 3. Results and Discussion

**Figure 1:**
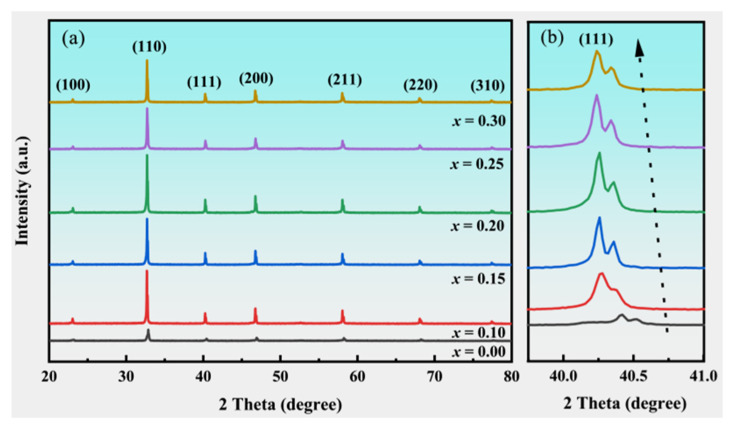
(**a**) Room-temperature XRD patterns of (1−*x*)BNBTM-*x*ST ceramics (0 ≤ *x* ≤ 0.30) over the 2θ range of 20°–80°. (**b**) High-resolution XRD patterns around the (111) reflection: 2θ = 39.5°–41°.

Figure 1a illustrates the 2θ-dependent XRD profiles (20°–80°) collected at room temperature for (1−*x*)BNBTM-*x*ST ceramics. According to the XRD measurements, all compositions exhibit a single-phase perovskite structure. No secondary phases are observed, indicating that ST is fully incorporated into the BNBTM lattice. As shown in the high-resolution XRD scan (Figure 1b), the (111) reflection gradually moves toward lower 2θ as the ST concentration increases. This peak shift originates from substituting smaller A-site cations (*R*_Bi_^3+^ = 0.136 nm, *R*_Na_^+^ = 0.139 nm) by larger Sr^2+^ (*R*_Sr_^2+^ = 0.144 nm), which enlarges the unit cell volume [23].

**Figure 2:**
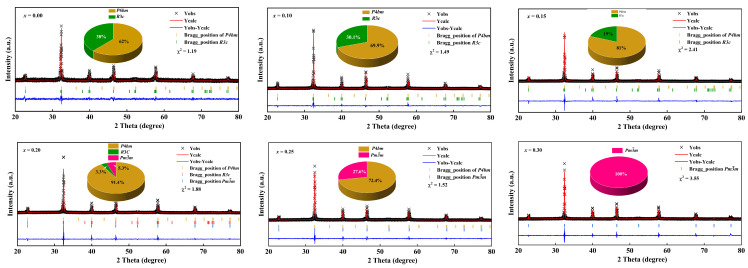
XRD patterns and Rietveld refinement profiles of (1−*x*)BNBTM-*x*ST (0 ≤ *x* ≤ 0.30) ceramics. The inset displays the refined phase fractions of the rhombohedral (*R*3*c*), tetragonal (*P*4*bm*), and cubic (*Pm*$\bar{3}$*m*) phases.

To further explore the observed phase evolution, XRD Rietveld refinement and phase recognition were performed on all (1−*x*)BNBTM-*x*ST samples (Figure 2), and the obtained lattice parameters as a function of *x* are summarized in Table 1. For the BNBTM sample, Rietveld refinement confirms the coexistence of *R*3*c* and *P*4*bm* phases, with the *P*4*bm* phase dominating at a fraction of 62%, exceeding that of the *R*3*c* phase (38%). For the compositions with *x* = 0.10 and 0.15, the *R*3*c* and *P*4*bm* phases still coexist. However, with increasing ST substitution, the *P*4*bm* phase progressively accumulates whereas the *R*3*c* phase counterpart diminishes. At *x* = 0.15, the *P*4*bm* phase fraction increases to 81%, whereas the *R*3*c* phase fraction decreases to 19%. Upon further doping to *x* = 0.20, the *P*4*bm* fraction reaches 91.4%, while a cubic (*Pm*$\bar{3}$*m*) phase concurrently emerges. At *x* = 0.25, the *R*3*c* phase disappears, and the *P*4*bm* fraction decreases to 72.4%, whereas the cubic (*Pm*$\bar{3}$*m*) phase increases to 27.6%. When the ST content further increases to *x* = 0.30, Rietveld refinement reveals an exclusively cubic phase, indicating complete stabilization of the ER state at this doping level.

**Figure 3:**
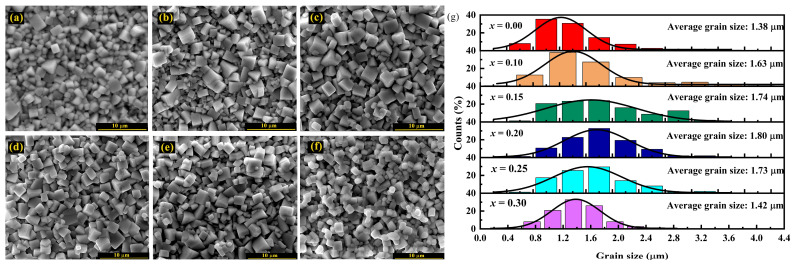
(**a**–**f**) SEM micrographs of (1−*x*)BNBTM-*x*ST ceramics as a function of *x* (0, 0.1, 0.15, 0.2, 0.25, 0.3). (**g**) Corresponding grain size statistics: distribution and average value.

**Figure 4:**
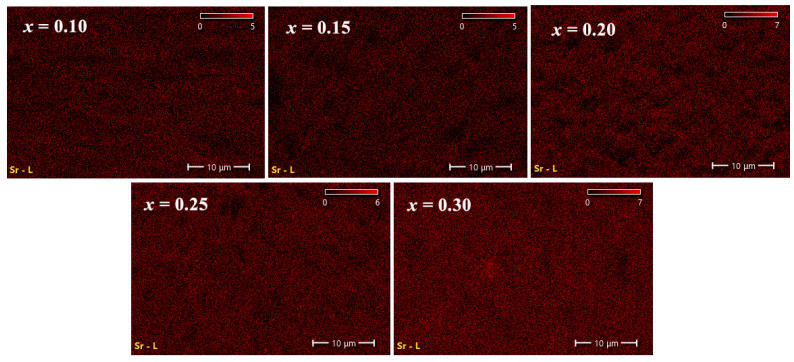
Sr elemental maps of (1−*x*)BNBTM-*x*ST ceramics.

The SEM micrographs in Figure 3a–f show that all (1−*x*)BNBTM-*x*ST ceramics exhibit well-defined grains and grain boundaries with no visible porosity. Figure 4 shows the Sr elemental maps of (1−*x*)BNBTM-*x*ST ceramics, revealing that Sr is uniformly distributed throughout the ceramics. Figure 3g presents the corresponding size histograms along with the estimated average grain size (AGS), derived using Nano Measurer (Version 1.2.0, Fudan University, Shanghai, China) from SEM micrographs. The AGS initially increases from 1.38 μm for BNBTM-0ST to a maximum of 1.80 μm for BNBTM-0.2ST, accompanied by a more uniform grain size distribution. This initial growth is likely promoted by accelerated mass transport following the introduction of ST [24]. When the ST concentration reaches *x* = 0.30, however, the AGS decreases to 1.42 μm. This reduction is attributed to a greater amount of Sr distributed at the grain boundaries, reducing the driving force for grain boundary migration and thus suppressing grain growth, which leads to a refined microstructure, consistent with previous findings [25].

**Figure 5:**
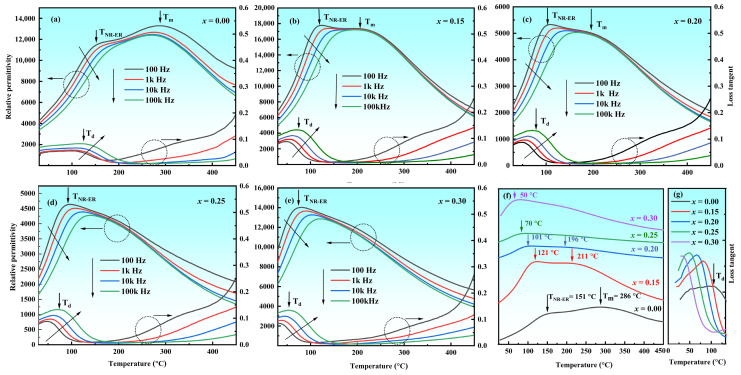
(**a**–**e**) Thermal-dielectric spectrum encompassing the relative permittivity (*ε*_r_) and loss tangent (tan*δ*) for (1−*x*)BNBTM-*x*ST ceramics (0 ≤ *x* ≤ 0.30) swept from 100 Hz to 100 kHz (the arrows indicate the direction of increasing frequency). (**f**,**g**) Temperature-induced evolution of *ε*_r_ and tan*δ* extracted from the 100 Hz.

**Figure 6:**
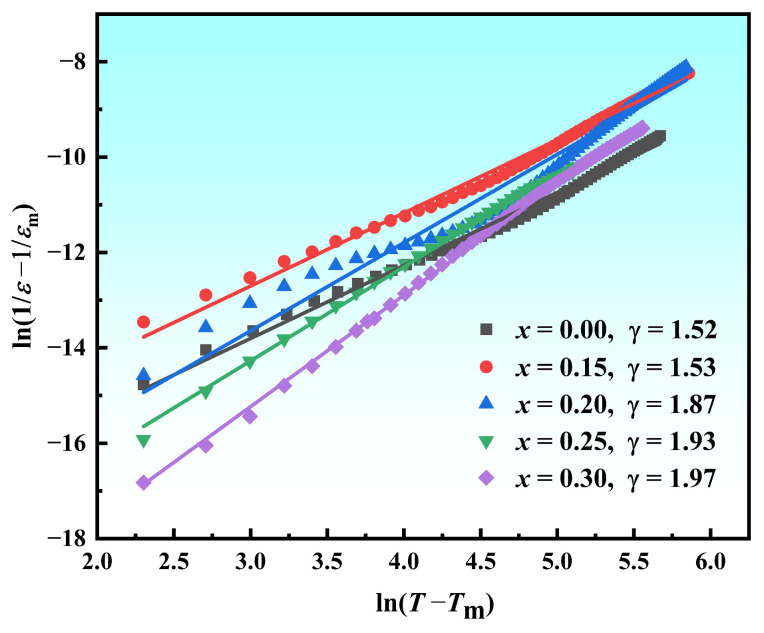
Linearized plot of ln(1/*ε* − 1/*ε*_m_) as a function of ln(*T* − *T*_m_) for (1−*x*)BNBTM-*x*ST ceramics, employed to extract the diffuseness (*γ*) via the modified Curie–Weiss law.

The broadband dielectric response, encompassing relative permittivity (*ε*_r_) and loss tangent (tan*δ*) as a function of temperature, is illustrated in Figure 5a–e for the (1−*x*)BNBTM-*x*ST ceramics (*x* = 0–0.3) from 100 Hz to 100 kHz. The upper curves represent *ε*_r_ and the lower curves represent tan*δ*. All compositions exhibit significant frequency dispersion, a defining characteristic of RFEs such as Pb(Mg_1/3_Nb_2/3_)O_3_ [26]. The high relative permittivity further highlights the potential of this system for energy storage applications [27]. The *ε*_r_-*T* curves for pure BNBTM (Figure 5a) exhibit two dielectric anomalies: a dielectric maximum near 286 °C (*T*_m_), marking the ferroelectric-to-paraelectric transition, and a pronounced step-like anomaly at a lower temperature (*T*_NR-ER_), indicating the NR-to-ER transition. As the ST concentration rises, the *T*_m_ peak moves monotonically to lower temperatures, while its intensity progressively weakens relative to the *T*_NR-ER_ anomaly (Figure 5b–f). For compositions with *x* ≥ 0.25 (Figure 5d,e), the *T*_m_ anomaly vanishes, evolving into a platform, while the *T*_NR-ER_ anomaly becomes fully dominant. Concurrently, similar to *T*_m_, the *T*_NR-ER_ anomalies shift to lower temperatures with increasing ST content (Figure 5f). The depolarization temperature (*T*_d_), identified via the tan*δ*-*T* peak (Figure 5a–e), marks the NR-to-ER transition [28,29,30]. With increasing ST content, *T*_d_ systematically decreases, falling to near room temperature at *x* = 0.30 (Figure 5g). The *T*_NR-ER_ anomaly originates from the thermal activation of polar nanoregions (PNRs), which drive the formation of the ER state [31,32]. The progressive intensification of this anomaly with increasing *x* suggests that ST doping enhances PNR dynamics, thereby effectively stabilizing and promoting the ER state. This evolving phase behavior is quantified by the diffuseness degree (*γ*), determined from the modified Curie–Weiss law [32,33,34]. As shown in Figure 6, *γ* displays an upward trend from 1.52 to 1.97 as *x* increases from 0 to 0.30, indicating a significant enhancement of relaxor diffuseness [35]. Taken together, the systematic increase in *γ*, accompanied by the drop in all characteristic temperatures (*T*_d_, *T*_NR-ER_, and *T*_m_), provides clear evidence that ST doping effectively stabilizes the ER state.

**Figure 7:**
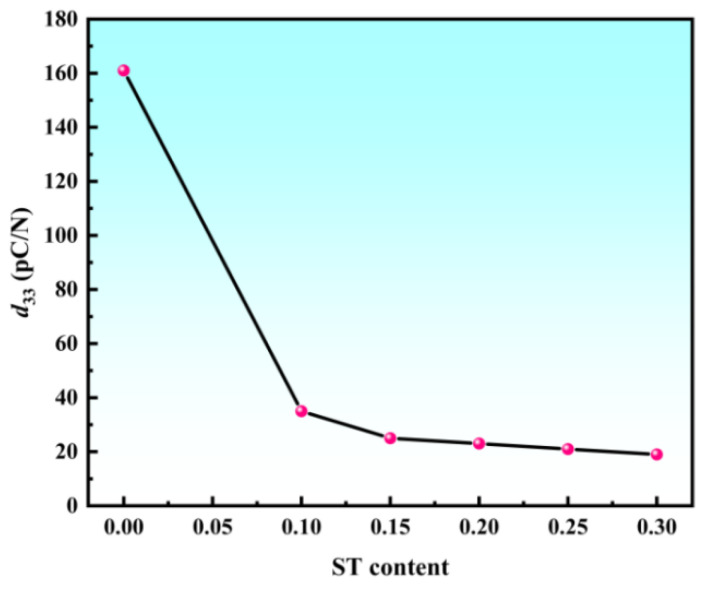
Piezoelectric coefficient (*d*_33_) versus ST content (*x*) for the poled (1−*x*)BNBTM-*x*ST ceramics (0 ≤ *x* ≤ 0.30).

The phase evolution of (1−*x*)BNBTM-*x*ST ceramics is directly reflected in their piezoelectric properties. As plotted in Figure 7, the piezoelectric coefficient (*d*_33_) reaches a maximum of 160 pC/N for pure BNBTM, followed by a sharp decline to approximately 35 pC/N at *x* = 0.10. With a further increase in ST content, *d*_33_ continues to decline, dropping to as low as 19 pC/N at *x* = 0.30. The high *d*_33_ in pure BNBTM indicates a predominantly NR state, which facilitates a strong piezoelectric response. The monotonic decrease in the *d*_33_ value with increasing ST content reflects a progressive NR-to-ER transition. The negligible *d*_33_ at *x* = 0.30 confirms that the ER state is predominantly stabilized under ambient conditions. This behavior is attributed to the suppression of the depolarization temperature *T*_d_, which is reduced to near-room temperature, thereby stabilizing the ER state under ambient conditions [26,36].

**Figure 8:**
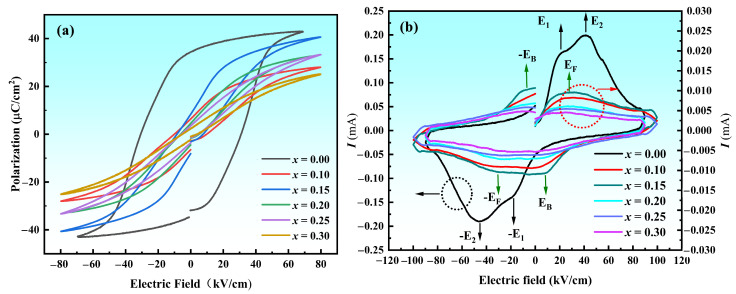
Ambient–temperature profiles of (**a**) polarization–electric field (*P*-*E*) hysteresis measured at 80 kV/cm, and (**b**) current–electric field (*I*-*E*) characteristics recorded up to individual near-breakdown fields for (1−*x*)BNBTM-*x*ST (0 ≤ *x* ≤ 0.30) ceramics.

**Figure 9:**
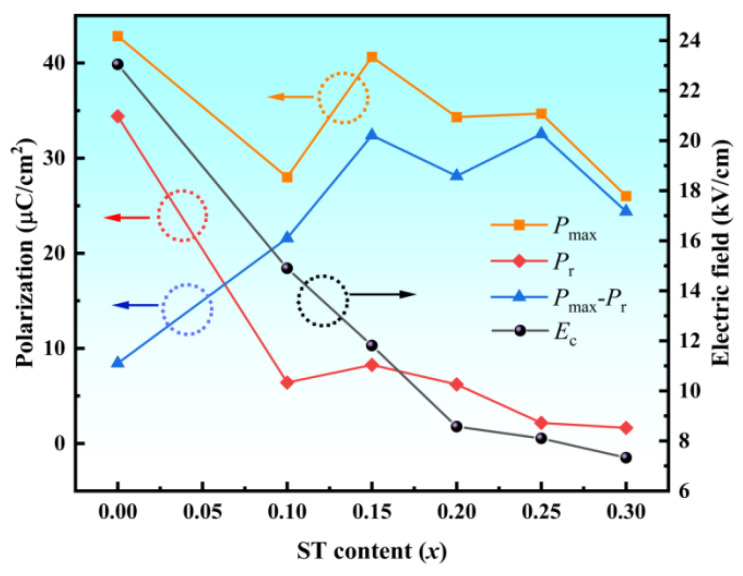
Composition-dependent ferroelectric parameters (*P_max_*, *P_r_*, Δ*P* and *E*_c_) of (1−*x*)BNBTM-*x*ST ceramics (0 ≤ *x* ≤ 0.30).

To investigate the composition-dependent ferroelectric performance in greater detail, we present the *P*-*E* hysteresis loops (Figure 8a) and associated current–electric field (*I*-*E*) curves (Figure 8b) of (1−*x*)BNBTM-*x*ST ceramics. As shown in Figure 8a, the pure BNBTM exhibits a well-saturated, square-like *P*-*E* loop. The loop features a high *P_m_* of 43 μC/cm^2^, a large *P*_r_ of 34 μC/cm^2^ and a moderate coercive field (*E*_c_) of 23 kV/cm (as summarized in Figure 9). Correspondingly, its *I*-*E* curve displays four distinct current peaks at ±E_1_ and ±E_2_. Under an increasing electric field, a gradual growth of PNRs occurs at ±E_1_, and subsequently, the macroscopic ferroelectric (FE) domains reorient at the higher electric field ±E_2_. Upon removal of the external bias, the polarization configurations established at +E_2_ and −E_2_ are recovered during subsequent field reversal at −E_1_ and +E_1_, accompanied by P = 0 in the intervening current valleys. This indicates that the FE domains formed at high electric fields do not revert to the initial PNR state upon field removal [37,38]. The high *P*_r_ and square-like *P*-*E* hysteresis loop (Figure 8a) confirm that this field-induced FE phase is sustained after unloading, indicating that the pure BNBTM resides in an NR state due to an irreversible transition from FE domains to a PNR state. For *x* = 0.15, despite a stable high *P_m_* (40.7 μC/cm^2^), both *P*_r_ and *E*_c_ drop sharply to 8.2 μC/cm^2^ and 14.9 kV/cm, respectively. The resulting *P*-*E* loop becomes visibly slender, indicating that ST disrupts the long-range ferroelectric order. The corresponding *I*-*E* curve in Figure 8b reveals that the polarization induced at ±E_F_ is recovered upon unloading at ±E_B_ and leads to a significant reduction in *P_r_* under zero electric field. This implies the metastability of such field-coerced long-range alignment, which spontaneously relaxes back into the PNR state upon the removal of the external bias. The reversibility of the ER-FE transition arises from the near-equivalence of free energy between the two states [39]. With increasing ST content, *P_m_*, *P*_r_, and *E*_c_ gradually decrease to negligible values, and the *I*-*E* peak intensity weakens until it virtually disappears. This behavior reflects the suppression of the initial NR phase and the subsequent stabilization of the ER state under zero-field conditions, demonstrating a compositional evolution from an NR-dominant to an ER-dominant state with increasing ST content [40,41].

**Figure 10:**
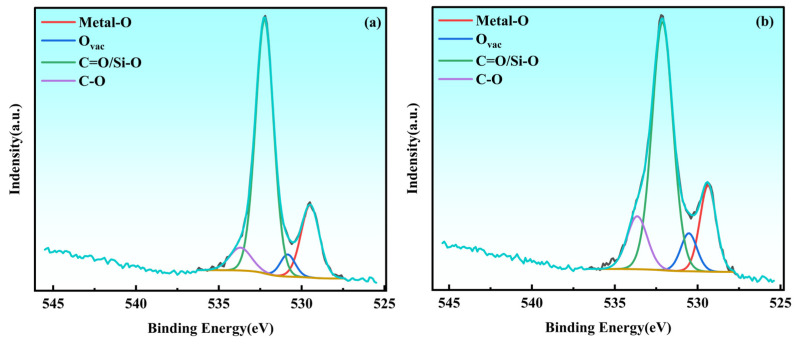
XPS O 1*s* core-level spectra for (1−*x*)BNBTM-*x*ST ceramics: (**a**) *x* = 0 and (**b**) *x* = 0.15.

The defects induced by Sr^2+^ doping also promote the transition of the samples from the NR to the ER relaxor state. XRD analysis shows that Sr^2+^ ions predominantly occupy the A-sites of the BNBTM lattice, substituting for Bi^3+^ and Na^+^. When Sr^2+^ replaces Bi^3+^, a local electrical imbalance is created, and additional oxygen vacancies form to neutralize the negative excess charge: $SrO\overset{{Bi}_{2}O_{3}}{\to }{Sr}_{Bi}^{\prime }+\frac{1}{2}V_{\ddot{O}}+O_{O}^{\times }$. When Sr^2+^ replaces Na^+^, an effective positive charge is introduced, which could theoretically be compensated by A-site vacancies ($V_{A}^{\prime }$). However, in BNT-based systems, the pronounced volatility of the A-site cations (Bi^3+^/Na^+^) under thermal processing primarily favors oxygen vacancy ($V_{\ddot{O}}$) compensation. Therefore, the oxygen vacancy concentration is expected to increase progressively with increasing Sr content. These $V_{\ddot{O}}$ can form defect dipoles with Ti^3+^ (e.g., Ti^3+^—$V_{\ddot{O}}$). The random fields introduced by these defect dipoles disrupt the long-range ferroelectric alignment, driving the NR-to-ER phase evolution. This defect mechanism was further substantiated by the O 1*s* photoemission features (Figure 10), probed via X-ray photoelectron spectroscopy (XPS). For the sample *x* = 0, the fitted peak associated with oxygen vacancies appears at a binding energy of 530.5 eV, comprising 4.89% of the total oxygen species (Figure 10a). This translates to an absolute lattice oxygen vacancy content of 1.37%, factored against the total oxygen elemental proportion (28.13%). By comparison, the *x* = 0.15 composition exhibits an elevated absolute oxygen vacancy content of 1.90% (Figure 10b). This clear concentration increment upon Sr^2+^ doping explicitly rationalizes the destabilization of the long-range ferroelectric alignment, triggering a pinched *P*-*E* loop and prompting the NR-to-ER transition.

**Figure 11:**
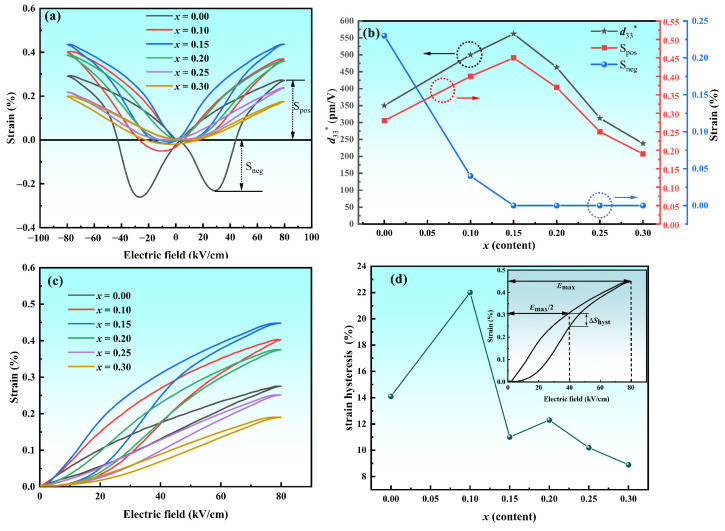
Electromechanical strain response of (1−*x*)BNBMT-*x*ST ceramics (0 ≤ *x* ≤ 0.30): (**a**) Bipolar *S*-*E* curves. (**b**) Composition-sensitive normalized strain (*d*_33_*), positive strain (*S*_pos_) and negative strain (*S*_neg_). (**c**) Unipolar *S*-*E* curves. (**d**) Strain hysteresis (*H*) versus ST content.

The electric-field-induced strain behaviors of (1−*x*)BNBTM-*x*ST ceramics are depicted in Figure 11a and Figure 11c, corresponding to the bipolar and unipolar (*S*-*E*) modes, respectively. Pure BNBTM exhibits a characteristic butterfly-shaped loop with a moderate positive strain (*S*_pos_) of 0.28% and a significant negative strain (*S*_neg_) of 0.23% (Figure 11b). The corresponding unipolar *S*-*E* loop is nearly linear, with a strain comparable to its bipolar positive counterpart. The butterfly-shaped bipolar strain curves along with the linear unipolar characteristics of pure BNBTM further confirm the presence of NR phase in BNBTM at room temperature, which is attributable to its *T*_NR-ER_ (~151 °C) being far above room temperature [37]. Upon ST doping to *x* = 0.15, both *S*_pos_ and *d*_33_* increase significantly, reaching optimal values of 0.45% and 562 pm/V, respectively, while *S*_neg_ progressively decreases to nearly zero. This evolution transforms the bipolar *S*-*E* curves from a butterfly to a sprout-like shape. Combining structural and polarization analyses, the progressive incorporation of ST induces a zero-field phase crossover from the NR to the ER relaxor state. At the optimal composition (*x* = 0.15), the free energies of the NR and ER phases are comparable, leading to a reduced energy barrier, thereby promoting domain switching and consequently maximizing the reversible strain [42]. Beyond *x* = 0.15, further ST addition leads to a progressive reduction in strain and *d*_33_* due to the deviation from the critical NR/ER phase ratio, which widens the free energy gap between the ER and FE phases. Meanwhile, the *S*_neg_ becomes negligible and the *S*-*E* curves gradually narrow, signifying enhanced relaxor behavior and further stabilization of the ER state [43]. Concurrently, the *H* initially increases with ST content and begins to decline at *x* ≥ 0.1, reaching a minimum of 8.9% at *x* = 0.30 (Figure 11d). This overall reduction in *H* is attributed to the increasing dominance of the ER state upon ST doping. For the *x* = 0.30 composition, the NR phase completely transforms to the ER phase, and the strain response originates from the activities of local PNRs.

Despite promising field-induced phase transitions for capacitor applications, BNT-based ceramics suffer from relatively low *W*_rec_ and *η* under low electric field, hampering their practical use in integrated circuits and wearable devices [44]. Theoretically, a maximized polarization span (Δ*P = P*_max_ − *P*_r_), together with enhanced dielectric breakdown strength (BDS) and minimized hysteresis loss, dictates the optimization of both *W*_rec_ and *η* [45]. In our (1−*x*)BNBTM-*x*ST system, increasing ST promotes the ER state, as confirmed earlier. This promotion of the ER state brings a significant reduction in *P*_r_ (thereby increasing Δ*P*) and a strongly pinched *P*-*E* hysteresis loop, originating from the activities of local PNRs. Benefiting from these synergistic features toward energy-storage capabilities, the preferential *x* = 0.25 composition delivers a maximum Δ*P* of 32.53 μC/cm^2^ alongside a highly constricted polarization profile (Figure 8a and Figure 9), suggesting enhanced energy storage performance at this composition.

**Figure 12:**
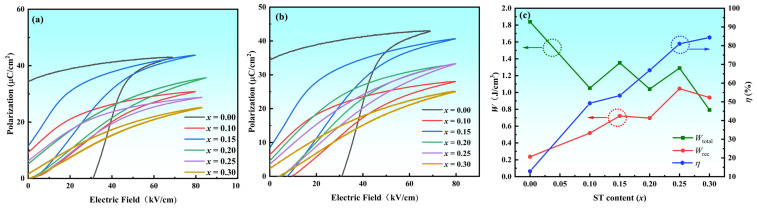
Ambient-temperature energy-storage characteristics for (1−*x*)BNBTM-*x*ST ceramics (0 ≤ *x* ≤ 0.30): unipolar *P*-*E* hysteresis loops evaluated at (**a**) individual near-breakdown strengths and (**b**) a uniform 80 kV/cm, together with (**c**) compositional evolution of *W_total_*, *W*_rec_ and *η*.

Figure 12a and Figure 12b present the evolution of unipolar *P*-*E* hysteresis loops with composition in (1−*x*)BNBTM-*x*ST ceramics measured near the breakdown strength and at 80 kV/cm, respectively. With increasing ST doping, the polarization hysteresis undergoes a progressive narrowing, a hallmark of enhanced relaxor characteristics triggered by the structural-distortion-driven formation of PNRs. Figure 12c summarizes the composition-dependent energy storage properties (*W*_total_, *W*_rec_, and *η*) of the (1−*x*)BNBTM-*x*ST ceramics (0 ≤ *x* ≤ 0.30) measured at 80 kV/cm, the maximum common field applicable to all compositions. As anticipated, under a low electric field of 80 kV/cm, the optimized *x* = 0.25 composition delivers a maximum *W*_rec_ of 1.06 J/cm^3^ and a competitive *W*_rec_/*E* of 0.013 mC/cm^2^, along with an impressive *η* of 81%. With further ST doping, the ER behavior becomes more pronounced, leading to a progressive decline in *P*_max_ (Figure 9). Although this decline limits ΔP and consequently *W*_rec_, the *η* further increases to 85%. This improvement in *η* is attributed to the increasingly slimmer *P*-*E* hysteresis loops originating from the enhanced ER characteristics. For a direct comparison of the strain and energy storage properties of (1−*x*)BNBTM-*x*ST materials, the relevant data from the previous literature are listed in Table 2. The results demonstrate that our material shows competitive overall performance.

## 4. Conclusions

In summary, we demonstrate a synergistic enhancement of strain and energy storage properties in (1−*x*)BNBTM-*x*ST ceramics via co-engineering of A-site defects and the NR/ER phase boundary. The incorporation of Sr^2+^ at the A-site introduces cationic disorder and promotes the formation of PNRs, which significantly suppresses *P*-*E* hysteresis and enlarges the Δ*P*, thereby boosting both *W*_rec_ and *η*. Concurrently, ST doping stabilizes the critical ER/NR phase boundary near room temperature, leading to a significant improvement in electromechanical strain. Consequently, an excellent strain of 0.45% (*d*_33_* = 562 pm/V) with a low *H* of 10.8% is achieved at *x* = 0.15. Meanwhile, under a low electrical bias of 80 kV/cm, the *x* = 0.25 composition delivers a maximum *W*_rec_ value (1.06 J/cm^3^) alongside an elevated *η* of 81% and a competitive *W*_rec_/*E* of 0.013 mC/cm^2^. This work establishes a viable strategy for designing multifunctional dielectrics capable of simultaneously delivering large strain and high energy storage performance. It should be noted that, owing to current instrumental boundaries, a systematic tracking of temperature-mediated strain and energy-storing traits remains for future exploration; such investigation, along with fatigue endurance evaluations, will be comprehensively conducted next to validate the material’s viability in practical micro devices (e.g., MEMS).

## Figures and Tables

**Table 1:** Lattice parameters derived from Rietveld refinement for (1−*x*)BNBTM-*x*ST ceramics.

Comp.	Lattice Parameters (Å)				
*x*	Rhombohedral (*R*3*c*)		Tetragonal (*P*4*bm*)		Cubic (*Pm*$\bar{3}$*m*)
0.00	*a* = b = 5.5544 Å	c = 13.5136 Å	*a* = b = 5.5260 Å	c = 3.9099 Å	
0.10	*a* = b = 5.5568 Å	c = 13.5000 Å	*a* = b = 5.5254 Å	c = 3.9038 Å	
0.15	*a* = b = 5.5613 Å	c = 13.5000 Å	*a* = b = 5.5264 Å	c = 3.9035 Å	
0.20	*a* = b = 5.5413 Å	c = 13.5000 Å	*a* = b = 5.5256 Å	c = 3.9095 Å	*a* = b = c = 3.9119 Å
0.25			*a* = b = 5.5234 Å	c = 3.9092 Å	*a* = b = c = 3.9056 Å
0.30					*a* = b = c = 3.9081 Å

**Table 2:** Comparison of strain, hysteresis (*H*), recoverable energy density (*W*_rec_) and energy efficiency *η* among reported studies.

Materials	*E* (kV/cm)	*S*_max_ (%)	*H* (%)	*W*_rec_ (J/cm^3^)	*η* (%)	References
Mn-doped NBBST	90	0.24	~	1.06	82	[2]
BNT–SLBT	90	0.45	~	0.75	85	[9]
NBT-ST-*x*Mn	89	0.22	14	1.14	83	[8]
(Pb,Sm)(Zr,Sn,Ti)O_3_	200	0.63	~	1.74	~	[5]
BNBTM-*x*ST	80	0.45	10.8	1.06	81	This work

## Data Availability

The original contributions presented in this study are included in the article. Further inquiries can be directed to the corresponding authors.
